# Shaping zoonosis risk: landscape ecology vs. landscape attractiveness for people, the case of tick-borne encephalitis in Sweden

**DOI:** 10.1186/1756-3305-7-370

**Published:** 2014-08-15

**Authors:** Caroline B Zeimes, Gert E Olsson, Marika Hjertqvist, Sophie O Vanwambeke

**Affiliations:** Earth and Life Institute, Georges Lemaître Centre for Earth and Climate Research (TECLIM), Université Catholique de Louvain (UCL), GEOG, Place Louis Pasteur 3 bte L4.03.07, 1348 Louvain-la-Neuve, Belgium; Department of Wildlife, Fish, and Environmental Studies, Swedish University of Agricultural Sciences (SLU), Umea, Sweden; Swedish Institute for Communicable Disease Control (SMI), Stockholm, Sweden

**Keywords:** Tick-borne disease, Zoonoses, Vector-borne disease, Risk

## Abstract

**Background:**

In this paper, the hazard and exposure concepts from risk assessment are applied in an innovative approach to understand zoonotic disease risk. Hazard is here related to the landscape ecology determining where the hosts, vectors and pathogens are and, exposure is defined as the attractiveness and accessibility to hazardous areas. Tick-borne encephalitis in Sweden was used as a case study.

**Methods:**

Three boosted regression tree models are compared: a hazard model, an exposure model and a global model which combines the two approaches.

**Results:**

The global model offers the best predictive power and the most accurate modelling. The highest probabilities were found in easy-to-reach places with high landscape diversity, holiday houses, waterbodies and, well-connected forests of oak, birch or pine, with open-area in their ecotones, a complex shape, numerous clear-cuts and, a variation in tree height.

**Conclusion:**

While conditions for access and use of hazardous areas are quite specific to Scandinavia, this study offers promising perspectives to improve our understanding of the distribution of zoonotic and vector-borne diseases in diverse contexts.

## Background

Most emerging diseases are of zoonotic origin [1]. As they involve pathogens, hosts and, potentially vectors, zoonoses are complex disease systems and a challenge for public health. In this paper, concepts of risk assessment are applied to a vector-borne zoonotic disease in an innovative approach to untangle sources of risk. Risk assessment includes the identification of hazard and the characterization of exposure [2]. The hazard is any potential source of damage (e.g. radioactive radiation), while the exposure is the chance that populations will be in contact with the hazard (e.g. work in a nuclear power plant). In the context of zoonotic vector-borne diseases, we define hazard as the number of infected hosts or vectors in the environment. This is determined by ecological conditions allowing the hosts, vectors and pathogens to complete their life cycles and to overlap. Exposure concerns the degree to which humans get in contact with infected hosts/vectors. This largely relates to land use, including the ability and the attraction to access places where infected hosts/vectors are found. Many disease ecology studies focus on what is here defined as hazard. Exposure is more commonly addressed by the field of public health which often does not include landscape-related variables. However, the distribution of disease cases potentially results from the combination of both hazard and exposure, and therefore cannot be approached solely from the hazard angle. In this study, we attempt to distinguish between hazard and exposure by comparing the predictive power of three models that focus on different aspects of the landscape: a hazard model containing a set of variables found in the ecological literature, an exposure model containing a set of variables found in the touristic and public health literature and, a global model containing both sets of variables. Tick-borne encephalitis (TBE) in Sweden is used as a case study. TBE has already been well studied under the hazard angle (e.g. [3]) but less under the angle of exposure (which is emphasized in [4]).

TBE virus (TBEV) belongs to the family of Flaviviruses and the western subtype of TBEV is usually transmitted by *Ixodes ricinus* ticks [5]. Ticks pass through three active life stages (larvae, nymphs and adults) and need a blood meal to reach the following respective stage [6]. Transmission of TBEV among ticks occurs mainly during co-feeding, especially between uninfected larva and infected nymph feeding on rodents [7–9].

TBEV is of concern in Sweden, as the tick population has spread and the incidence of the disease has been increasing sharply over the past few years [10]. Two phenomena are currently observed in Sweden. On the one hand, the range of human cases of TBE is expanding westward within the known tick range, and on the other hand, the expansion of ticks northward along the coast. While some common factors may be at play, the mechanisms behind each phenomenon have not been fully clarified. This may result from climate changes, host populations dynamics and human behaviour changes [11, 12]. However, even in the well-established TBEV endemic areas around Stockholm, the effect of these variables on spatial distribution of the disease is unclear.

## Methods

### Materials

**Figure 1:**
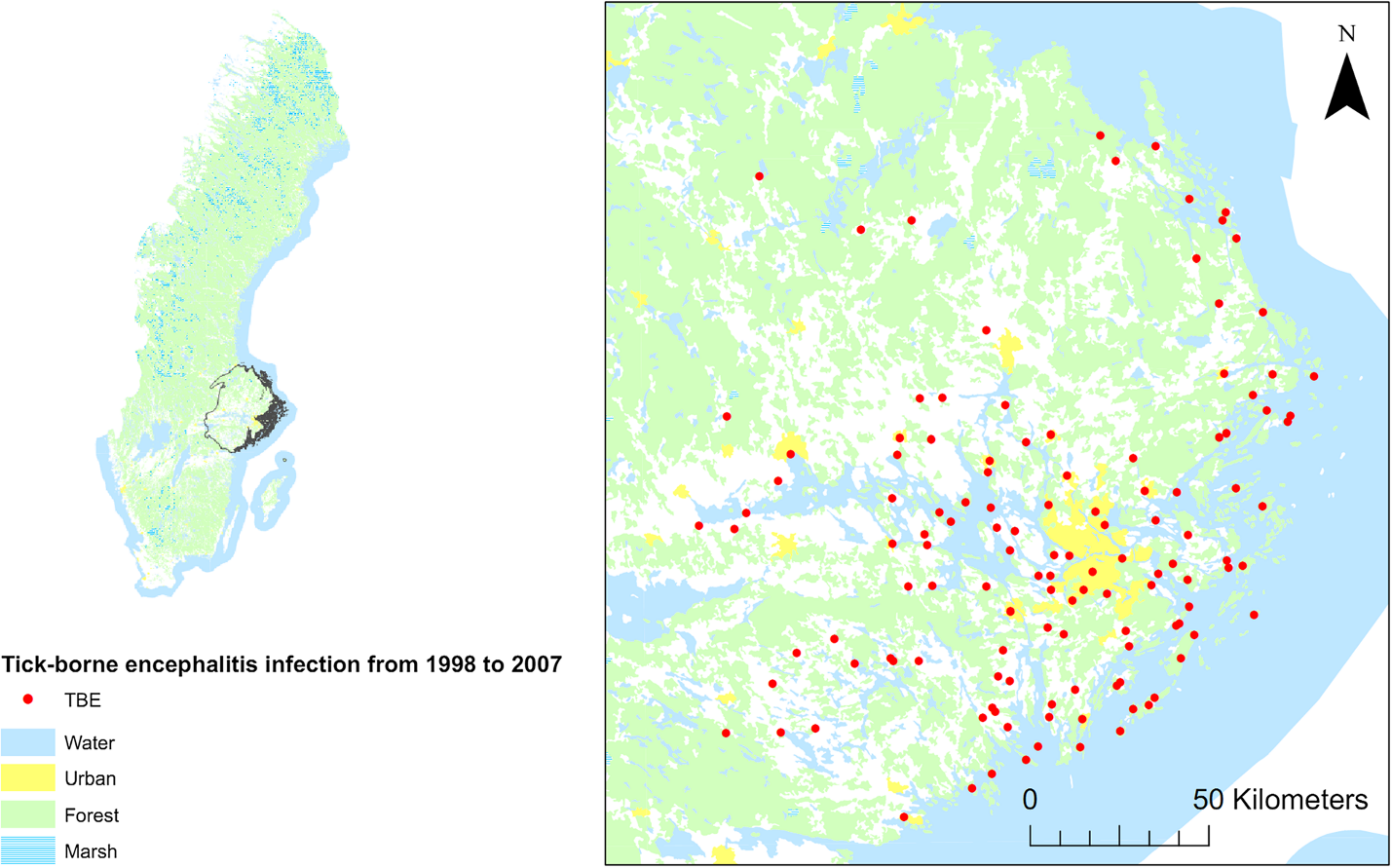
**Human infections of tick-borne encephalitis in Sweden.**

The study focused on Stockholm and the five neighbouring counties (Gävleborgs län, Dalarnas län, Uppsala län, Västmanlands län and Södermanlands län) (Figure 1). TBEV is well established in that region of Sweden, where the disease has been recorded for the past century. Records of cases by nearest settlement of infection (SMI Swedish Institute for Communicable Disease Control (Smittskyddsinstitutet) were included for a ten-year study period (January 1998 to December 2007). Presence at any time during the study period was translated into a presence record, totaling 125 presence records. The other settlements extracted from the Lantmäteriet database (Swedish mapping, cadastral and land registration authority), constituted the 4297 absence records.

Potential explanatory variables either represented the surrounding environment, as calculated in a radius of two km around the point location, or were calculated at the exact record location.

Hypotheses were made based on literature, and candidate explanatory variables were allocated to hazard or exposure. However, some variables could not be allocated to a single group in an unequivocal way and were included in both.

#### Variables describing hazard

Variables describing the hazard, places where infected hosts or vectors are found, were identified in the literature (Table 1). They are linked to the ecology of the hosts and vector. Some commonly used landscape metrics were also included.

**Table 1:** **Variables selected in the hazard or exposure model**

	Hazard	Exposure	Resolution	Units	Sources
Roe deer/reddeer/fallow deer/wild boar	X		Low (interpolation based on hunting districts centers)	Number of animals per hectar	Dr. Jonas Kindberg
Proportion of forest in the buffer	X	X	100 m	Percentage	CORINE
Proportion of broad-leaved forest in the buffer	X	X	100 m	Percentage	CORINE
Proportion of coniferous forest in the buffer	X		100 m	Percentage	CORINE
Proportion of mixed forest in the buffer	X		100 m	Percentage	CORINE
Shape index of forest in the buffer	X		100 m	None	CORINE
Mean proximity index for forest patches in the buffer	X		100 m	None	CORINE
Mean volume of spruce/pine/birch/oak in the buffer	X		30 m	m^3^/ha	SLU skogskarta
Proportion of clear-cuts (tree height < 50 cm) in the forest in the buffer	X	X	30 m	Percentage	SLU skogskarta
Proportion of waterbodies in the buffer	X	X	100 m	Percentage	CORINE
Distance to the nearest water course	X	X	High (shapefile)	m	Lantmäteriet
Proportion of open areas in ecotone of 150 m around forest in the buffer	X		100 m	Percentage	CORINE
Shannon diversity index in the buffer	X		100 m	None	CORINE
Length of roads in the buffer		X	High (shapefile)	m	Lantmäteriet
Length of roads in forest in the buffer		X	High (shapefile)	m	Lantmäteriet
Distance to Stockholm		X	High (shapefile)	m	Lantmäteriet
Proportion of area occupied by holiday houses in the buffer		X	High (shapefile)	Percentage	Statistiska Centralbyrån
Mean population density		X	2.5 arc-minutes	Person/km^2^	Gridded Population of the World
Distance to the sea		X	High (shapefile)	m	Lantmäteriet
Standard deviation of tree height in the buffer		X	30 m	m	SLU skogskarta
Mean height tree		X	30 m	m	SLU skogskarta

i. Animal species

In Sweden, roe deer are a major blood meal host for reproducing adult female ticks [11, 13]. However, other larger game species, e.g. red deer, fallow deer and wild boar, are available in large numbers and are also likely to be important hosts for ticks. Bag records of these game species were included (Dr Jonas Kindberg, Wildlife Monitoring Unit, Swedish Association for Hunting and Wildlife Management, personal communication). Data, available by centre of hunting districts, were interpolated by Thiessen polygons, leading to a lower resolution compared to the other variables. The number of animals per hectare found at the point is used as a proxy for blood meal availability.

ii. Forest

Deciduous forests are a highly suitable habitat for ticks, as well as for some host mammals [14, 15]. The total proportion of forest, broad-leaved forest, conifer and mixed forest inside the two km buffer (100 m resolution, CORINE Land Cover, EEA) were included. The average shape and proximity index of forest patches in the buffer were calculated. A patch with the most compact shape (i.e. the smallest patch to area ratio), in the case of raster data, a square, has a shape index of one. Increasing values indicate a more complex shape, and more contact between the patch and its surroundings. The proximity index of forest patches relates to the amount of forest within a specified radius around a patch, and indicates whether a patch is isolated or fragmented.

Also, as various tree species may impact tick habitat suitability differently, the mean volume of spruce, oak, birch and pine per hectare in the buffer were added (30 meter resolution, SLU Skogskarta, Swedish University of Agricultural Sciences).

Forest areas where tree height was lower than 50 centimeters were used as a proxy to represent clear-cuts (SLU Skogskarta). Intensive clear-cutting is non-valuable for wildlife but, in the study area, clear-cuts were small (mean area of 1682.68 m^2^ and mean cross-section of 146.54 m). The area of clear-cuts was divided by the area of forest in the buffer. While clear-cuts may provide food for various host species, it does not provide as good shelter as forests.

iii. Land cover

Forest ecotones, particularly where they connect to open areas, can be very suitable for ticks and hosts as these habitats offer a high diversity of resources [16]. The main roe deer habitat is also deciduous or mixed forest with open areas [13, 15]. The proportion of open areas (agricultural and transitional area from CORINE) in ecotones of 150 meters around forests in the buffer was added to the hazard model.

The Shannon diversity index, representing the richness of the landscape in the buffer, was included [17].

Since high humidity favours tick questing, moist areas are more suitable for ticks [16]. The proportion of waterbodies in the buffer (CORINE) and the distance to the nearest water course (Lantmäteriet) were included as proxies for moister areas.

#### Variables describing exposure

Variables describing the exposure, the degree to which people enter infected landscape, were identified through the scientific literature studying landscape attractiveness for touristic activities (Table 1).

i. Accessibility

A study of tourist preferences indicated that accessibility to forest increases the touristic value of forest [18]. Indeed, in Sweden, there is a traditional right of public access to private land, e.g. to enter forests and to harvest resources such as mushrooms and berries [18, 19]. Assuming that roads increase access, and that forests with roads are more likely to be entered by visitors, we included the length of roads in the buffer (Lantmäteriet) and the length of roads in forests in the buffer to describe accessibility.

Assuming that places with holiday cabins would relate to outdoor activities, the area occupied by holiday houses in the buffer was included (Statistics Sweden (Statistiska Centralbyrån)). In Sweden, 50 of holiday houses are within a radius of 32 kilometres from permanent homes [20]. The distance to Stockholm, from which many holiday cabin users originate, was included in the model (Lantmäteriet database), assuming that areas closer to Stockholm would be more frequently used for outdoor recreation. Population density (2.5 arc-minutes resolution, Gridded Population of the World from Center for International Earth Science Information Network (CIESIN)) was also included.

ii. Scenic beauty

Landscape features documented to increase the perceived scenic beauty include water features [21] and broad-leaved forests [18]. The distance to the nearest water course (Lantmäteriet database), proportion of waterbodies in the buffer (CORINE), and the proportion of forest and of broad-leaved forest (CORINE) were used. In Finland, a preference for forest stands with a higher mean tree height and a skewed distribution of height has been highlighted [22]. The mean tree height in the buffer was calculated and standard deviation of tree height was used as a proxy for the skewness (SLU Skogskarta). In Sweden, the touristic value of a forest increases with the number of clear-cuts and decreases with the size of the clear-cuts within a given area [18]. The proportion of clear-cuts in the forest (SLU Skogskarta) in the buffer was thus added to the exposure dataset.

### Methods

#### Principal component analyses

The potential explanatory variables outlined above are numerous, mostly proxies, and sometimes redundant. Therefore, principal component analyses (PCA) were used to identify sub-groups of similar variables (“Rcmdr” package and plugin “FactoMineR” in R 2.12.0). The factorial coordinates were used as new variables. Two variables were selected: one summarizing the variables on wildlife species (wild boar, red deer, fallow deer and roe deer) and another, accessibility variables (population density, length of roads, distance to Stockholm and length of roads in forest).

### Boosted regression trees

The multivariate models were built using boosted regression trees (BRT) (“gbm” package in R) [23]. BRT have been identified as an efficient method for investigating variables explaining the spatial distribution of zoonotic diseases [24]. A major advantage compared to regression is that BRT allows the modelling non-linear responses. BRT results comprises of two essential elements: relative importance and response curve. The relative importance of each variable represents the number of times a variable was used in successive trees, weighted by the mean of the squared improvement provided by this variable to each tree [23]. Response curves are graphs representing the evolution of the fitted probability function according to the variation of the variable. They were interpreted here as a relative probability of being in the presence of the disease at various levels of the predictor variable.

In a BRT, an internal node represents a variable that is cutting the data into several branches that lead to other nodes [23]. The decision of presence or absence is made at terminal nodes. The new trees are fitted on the residuals of the previous trees and the new model contains both previous and new trees. At each step, 50 of the data are randomly selected to enlarge the previous trees. The learning rate (contribution of each tree to the final model), the tree complexity (number of nodes in a tree) and the number of trees are chosen in order to optimize the predictive power.

Three models were built containing respectively the hazard variables, the exposure variables, and both hazard and exposure in a global model. Some variables were included in both hazard and exposure models as they may relate to either aspect (Table 1). To account for potential spatial structure in the distribution of TBE cases, the proportion of infected places within a radius of 20 km was added to each model.

### Measures of the predictive power

#### Internal validation of the predictive power

As BRT builds the trees on random subsamples, each model (hazard, exposure and global) was run 25 times. The mean areas under the curve (AUC) (“PresenceAbsence” package in R) were compared using a Student t-test. An AUC of 0.5 indicates a random distribution of predictions, and of 1 a perfect prediction [25, 26]. False presences and absences, using the sensitivity equals the specificity as probability threshold, were mapped.

#### External validation of the predictive power

External AUC for each model were calculated from cross-validation on 10 subsets. Models were run on nine subsamples and AUC was calculated on a tenth sub-sample. This step is repeated 10 times, using a different validation sub-sample each time. The final AUC is the mean of the AUC calculated on the 10 evaluation sub-samples. This was run 25 times and compared with a Student t-test.

Moreover, TBE records from 2011 were used to assess the predictive power of our models. Continuous predicted probability maps were created for each model by kriging, on which TBE presence records in 2011 were overlaid.

The presence in 2011 were completed with absences (settlements with no presence records between 1998–2007 and in 2011). Then, predicted probabilities were calculated for this new dataset. The means of predicted probabilities located at presence points were compared to absences points by a Welch test.

## Results

### Principal component analyses

Two PCAs were computed. The two first components of the PCA on the data on wildlife species explained 71.76 of the variance. The first component (variable PC1: Wildlife) was positively correlated with wild boars (correlation of 0.85), red deer (0.84) and fallow deer (0.64). The second component was only positively correlated with roe deer (0.99), which was subsequently kept as an individual variable. The first two components of the PCA on accessibility variables explained 66.36 of the variance. The first component (variable PC1: Accessibility) was positively correlated with the human population density (correlation of 0.76), the length of roads (0.65) and negatively correlated with the distance to Stockholm (-0.75). The second dimension was positively correlated with the length of roads in the forest (0.92) and was also kept as an individual variable.

### Boosted regression trees

The variables with the highest relative importance (variables forming the first 50 of summed relative importance) in the hazard model were the number of TBE cases within 20 km (relative importance of 23.23), volume of spruce (10.71), distance to a water course (7.30), total proportion of forest (5.40) and proportion of coniferous forest (5.33) (Table 2).

**Table 2:** **Relative importance of variables introduced in the hazard and in the exposure boosted regression trees**

Hazard model		Exposure model	
Variable	Relative importance (%)	Variable	Relative importance (%)
Infections in 20 km	23.23	Infection in 20 km	25.42
Volume of spruce	10.71	Length of roads in forest	17.67
Distance to water course	7.30	Distance to water course	7.79
Volume of oak	6.33	Proportion of forest	7.75
Proportion of forest	5.40	Mean height of trees	7.09
Proportion of coniferous	5.33	PC1: Accessibility	6.78
Proportion of clear-cuts	5.28	Proportion of holiday houses	6.18
Volume of birch	5.24	Distance to the sea	5.49
Forest shape index	5.04	Standard deviation of height of trees	5.19
Shannon diversity index	4.83	Proportion of clear-cuts	5.19
Volume of pine	4.47	Proportion of broad-leaved forest	3.74
Proportion of mixed forest	4.26	Proportion of waterbodies	1.72
Forest proximity index	3.60		
Open areas in ecotones	3.05		
Proportion of broad-leaved forest	2.20		
PC1: Wildlife	1.31		
Proportion of waterbodies	1.31		
Roe deer	1.05		

In the exposure model, the variables with the highest relative importance were the number of TBE cases within 20 km (relative importance of 25.42), length of roads in forest (17.67) and distance to a water course (7.79) (Table 2).

**Figure 2:**
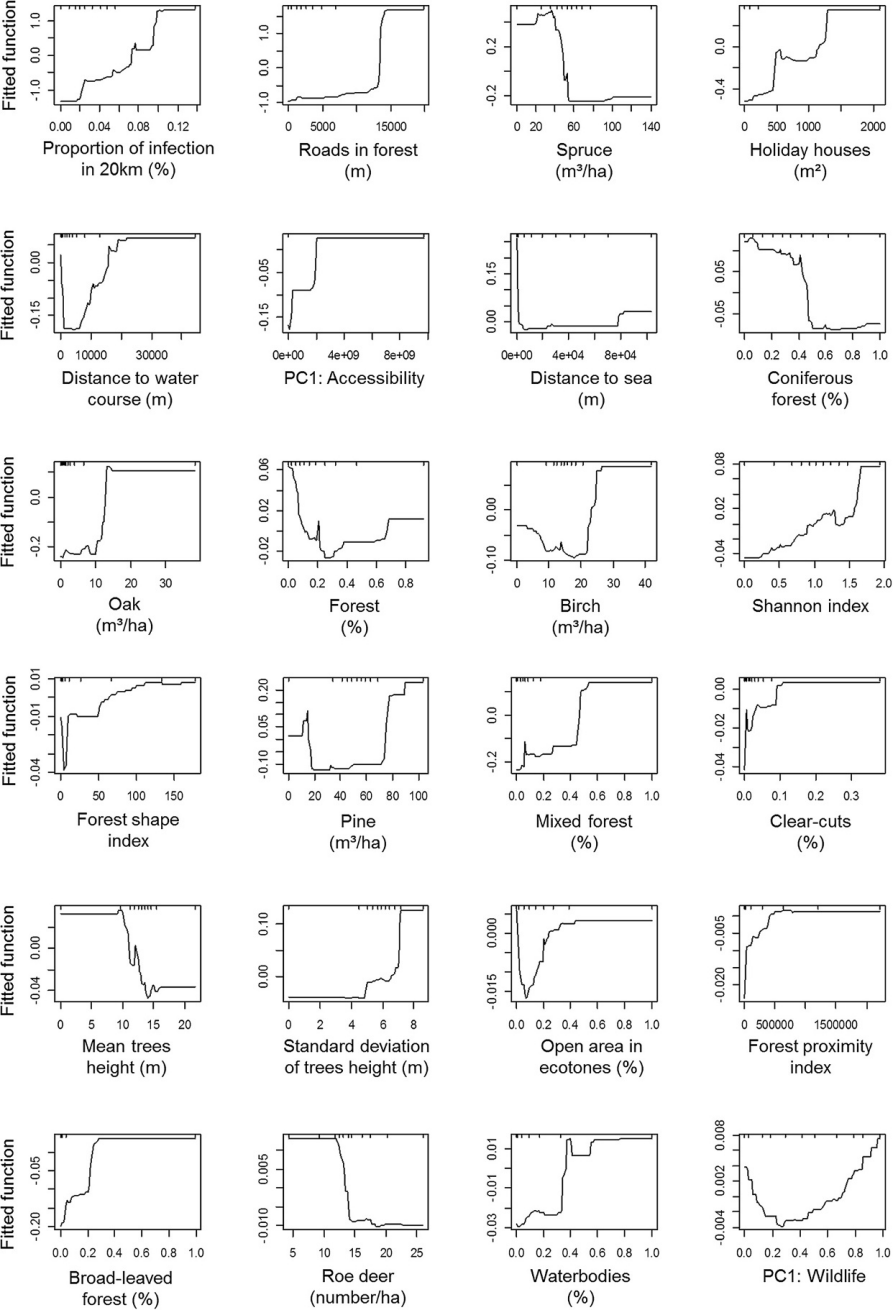
**Graphs of each variable according to the fitted function of the global model (percentage represents the relative importance of the variable).**

The trends of the variable response curve according to the probability of finding TBEV were similar in the tree models. Response curve graphs of the global model are represented in Figure 2. Variables which showed a global positive trend are: infections in 20 km (relative importance of 17.94); roads in forest (11.56), holiday houses (4.94), PC1: Accessibility (4.16), oak (3.76), birch (3.50), Shannon index (3.19), forest shape index (3.17), mixed forest (3.00), clear-cuts (2.88), standard deviation of tree height (2.71), forest proximity index (2.07), broad-leaved forest (1.52) and, waterbodies (0.97). Variables that showed a global negative trend are: spruce (8.10), coniferous (3.84), mean trees height (2.78) and roe deer (1.35). Variables which showed an important decrease followed by an increase are: distance to water course (4.55), distance to the sea (4.15), forest (3.70), open area in ecotone (2.31) and, PC1: Wildlife (0.74). Pine (3.12) showed a first peak around 15 m^3^/ha followed by an increase around 70 m^3^/ha.

### Measures of the predictive power

**Figure 3:**
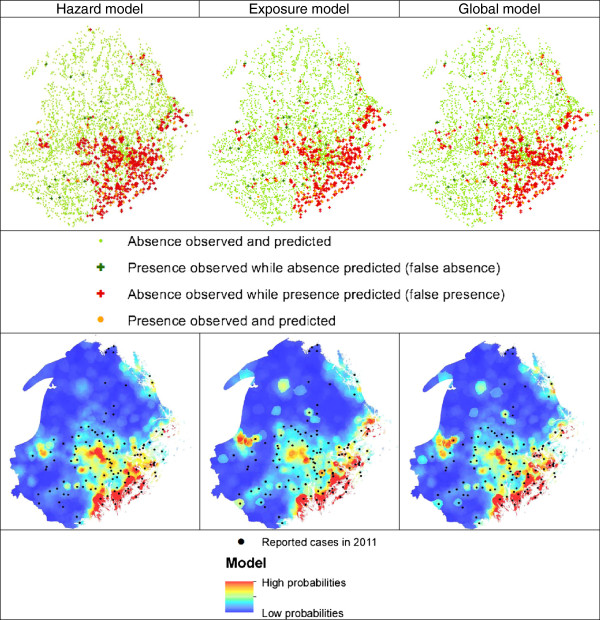
**Resulting maps of the hazard, exposure and global models (based on 1998–2007 TBE records) and TBE records in 2011.**

The mean AUC were 0.92 for the hazard and exposure models and 0.93 for the global model. AUC were lower for the cross-validated data with 0.74 for the hazard and exposure models and 0.75 for the global model. The mean AUC of global models are significantly higher (p-value <0.001) than the mean AUC of hazard models and exposure models.There were few false absences: 18 for the hazard model, 30 for the exposure model and 18 for the global model (Figure 3). There were more false presences: 681 for the hazard model, 482 for the exposure model and 593 for the global model (Figure 3).The false presence of the hazard models were in areas within the TBEV focus while false presences of exposure model were distributed in areas where disease cases have not yet been recorded (Figure 3). The global model had the best visual match between areas with high interpolated probabilities and high frequency of presence in 2011. The hazard and exposure models both appear to contribute to the distribution of high probability areas observed in the global model map (Figure 3).

The mean probability predicted by the global model on presence in 2011 (44.05*10^-3^) was significantly different (p-value < 0.001) from the mean probability of absence (27.51*10^-3^). Similar results were observed for probabilities extracted from the hazard and exposure models.

## Discussion

### Comparison of hazard and exposure

All three models, focusing on hazard, exposure and all factors, respectively, reached a good fit and a reasonable ability to predict hot spots for an independent year, i.e. 2011. However, the global model was clearly the most exhaustive in indicating areas of higher probabilities. Both the aspects of hazard and exposure therefore deserve consideration when examining risk and its spatial distribution. The two variables with the highest relative importance in the global model, other than the variable describing spatial structure, were the roads in forest positively related to the exposure, and the volume of spruce negatively related to the hazard, underlining the importance of accounting for both aspects of risk. This makes perfect sense when approaching the question of the spatial distribution of a zoonotic disease using human case records: human land use is spatially heterogeneous. Beyond the need to account for all factors explaining the spatial distribution, factors related to exposure may offer important keys for the understanding of zoonotic disease emergence and human risk. In this study, the spatial distribution of TBE cases in Sweden looked beyond hazard-related factors and classic epidemiologic factors such as occupation to include variables depicting specifically where people at risk are most likely to enter infested areas.False presences identified for the various models did not follow the same spatial pattern (Figure 3). These false presences could be: models errors; locations suitable for transmission but where the pathogen or susceptible humans are absent; areas where the pathogen is circulating but not transmitted to humans and; areas where the disease is found but not recorded (non-identified, non-reported or asymptomatic cases). Considering false presence as points were TBEV may appear in the near future, the exposure model seems suitable for predicting the disease in new areas. Inversely, looking at the probability maps, the hazard model seems to show a better prediction in areas of spatially concentrated TBE infectious areas and so a better prediction of intensification of the disease. There are still a few points outside the high probability areas, indicating that some variables may be missing in the models.

Challenges related to this hazard/exposure approach relate essentially to the interpretation of variables as influencing hazard or exposure. Some variables may be related to hazard or exposure in an unambiguous way, but several may be proxy for both vector (or host) habitat and landscape attractiveness for human. For example, while tree species would presumably relate to hazard, a study conducted in Finland indicates that touristic preferences increase with the volume of pines and birches [22]. Scale may influence the interpretation of variables as related to hazard or exposure. For example, the distance to the sea, here used in the exposure model, may also influence, at broader scale, the length of the vegetation season and the suitability for vectors and hosts through its buffering effect on temperature [14]. Careful consideration of the interpretation and scale of variables included in risk, hazard or exposure models is therefore necessary.

Both hazard and exposure variables are needed for a better understanding of the spatial distribution of vector-borne diseases, but exposure variables may be specific to regions, just as some epidemiological risk factors can be culturally driven. Sweden, for instance has a particular public right of access to land (“Allemansrätten”). Still, accessibility is not evenly developed everywhere, and other factors may influence accessibility or attractiveness, such as land ownership [27].

### Variables influencing the distribution of TBE in Sweden

This study highlights the main spatial variables influencing the distribution of TBE in a highly endemic region of Sweden. Forests with the highest probabilities of presence were well-connected oak, birch and pine forests, with a complex shape, numerous clear-cuts and a tree height variation of at least five meters. Landscapes with the highest probabilities were easy to reach, with high landscape diversity (Shannon index), holiday houses, waterbodies and broad-leaf or mixed forest with open area in their ecotones. Fitted function curves mostly follow our preliminary assumptions based on the literature or field experience. Therefore, spruce forests are less favourable than pine forests, probably because they have less undergrowth. Also, the high probability in very close proximity to the sea is related to the presence of houses near the shore. The results for the distance to water course raise new questions as it is a less prominent feature of the landscape than waterbodies.

Few interactions were identified. The most important interaction was between areas of holiday houses, a proxy for attractiveness, and places where there were more than 10 km of roads in forest, a proxy for accessibility. Furthermore, correlation between variables makes some probability distributions difficult to interpret. For instance, tree height, which is positively correlated to the proportion of coniferous trees, is negatively correlated to broad-leaved-forest. Decreasing probabilities with the tree height may thus relate to the decreasing probabilities with conifers. Also, low pine volume may imply the presence of larger volumes of deciduous trees and explain the peak around 17 m^3^/ha.

Probabilities of TBE cases increased with the first PCA component of wildlife (positively correlated with wild boars, red deer and fallow deer) and decreased with the abundance of roe deer. Deer and wild boars (and, maybe, more specifically, young wild boars) most likely constitute important blood meal sources for adult female ticks before egg laying. A negative response of TBE with roe deer was previously highlighted in Sweden [13] and, at a local scale, in Italy and Slovakia, with the increase of co-feeding ticks on rodent when deer density is decreasing [28]. Both studies hypothesized a dilution effect due to a high density of deer (incompetent hosts) diverting the questing ticks from rodents (competent hosts). However, a mathematical model estimating the threshold for tick-borne disease persistence reveals that, in the case of non-viraemic transmission, the dilution effect is less relevant [29]. Here, the PCA reflects that roe deer are not found in the same places as the three other species. The decreasing probability may therefore result not from roe deer specifically, but from unsuitability for any aspect of the transmission cycle. Further investigation on the role of wildlife in feeding ticks and hosting the TBE virus would be useful. These results highlight the need for a better understanding of the TBEV transmission system and the mechanisms underlying statistical relationship. Only in this way could such results be meaningful for risk prediction and public health.

## Conclusions

Our study of the distribution of human cases of TBE in Sweden indicates that separating and accounting specifically for hazard and exposure in distribution models holds great potential for the understanding and the mapping of zoonotic disease spatial pattern and emergence. Exposure variables were extracted from standard GIS data bases following a similar strategy as is classically done for studies focusing on the hazard.

TBE is emerging in different places in Europe and understanding this pattern is essential to help public health. Randolph compares human cases to “the tip of the iceberg” that emerges from the undetected enzootic cycles below the surface [3]. As ecological processes driving the distribution of TBEV are not yet completely described, it is of great value to be able to track the sources of human TBE back to infection sites, trying to unravel the role of local wildlife on the persistence and circulation of TBEV. Accounting for exposure may also contribute to this by allowing more specific interpretation of any variable in the model.

In conclusion, linking ecology and public health is highly recommended. While the conditions for access and use of hazardous areas highlighted in this study may be specific to Scandinavia, this unified method offers promising perspectives to further understand the distribution of various zoonotic and vector-borne diseases in diverse contexts by the explicit inclusion of exposure-related variables.

## Abbreviations

TBE: Tick-borne encephalitis
TBEV: Tick-borne encephalitis virus
PCA: Principal component analysis
BRT: Boosted regression trees
AUC: Area under the curve.
